# Post-Domestication Selection in the Maize Starch Pathway

**DOI:** 10.1371/journal.pone.0007612

**Published:** 2009-10-27

**Authors:** Longjiang Fan, Jiandong Bao, Yu Wang, Jianqiang Yao, Yijie Gui, Weiming Hu, Jinqing Zhu, Mengqian Zeng, Yu Li, Yunbi Xu

**Affiliations:** 1 Institute of Crop Science and Institute of Bioinformatics, Department of Agronomy, Zhejiang University, Hangzhou, China; 2 Institute of Crop Science, Zhejiang Academy of Agricultural Science, Hangzhou, China; 3 Institute of Genetics and Developmental Biology, Chinese Academy of Science, Beijing, China; 4 Institute of Crop Science, Chinese Academy of Agricultural Sciences, Beijing, China; 5 International Maize and Wheat Improvement Center (CIMMYT), Mexico, D.F., Mexico; University of Umeå, Sweden

## Abstract

Modern crops have usually experienced domestication selection and subsequent genetic improvement (post-domestication selection). Chinese waxy maize, which originated from non-glutinous domesticated maize (*Zea mays* ssp. *mays*), provides a unique model for investigating the post-domestication selection of maize. In this study, the genetic diversity of six key genes in the starch pathway was investigated in a glutinous population that included 55 Chinese waxy accessions, and a selective bottleneck that resulted in apparent reductions in diversity in Chinese waxy maize was observed. Significant positive selection in *waxy* (*wx*) but not *amylose extender1* (*ae1*) was detected in the glutinous population, in complete contrast to the findings in non-glutinous maize, which indicated a shift in the selection target from *ae1* to *wx* during the improvement of Chinese waxy maize. Our results suggest that an agronomic trait can be quickly improved into a target trait with changes in the selection target among genes in a crop pathway.

## Introduction

Modern crops have developed through artificial selection, which has usually included two evolutionary stages: domestication selection and subsequent genetic improvement (post-domestication selection). Previous studies have indicated that both selection events result in a loss of genetic diversity from a wild progenitor to its domesticated crop, and selected target genes, i.e. domestication or improvement genes, are expected to retain less diversity than neutral (unselected) genes, which are only impacted by bottleneck effects. Based on the amount of diversity and other sequence features (such as site frequency spectrum, linkage disequilibrium and population differentiation), it has become possible to search for selection *via* a population genetics approach [1]–[6].

**Figure 1:**
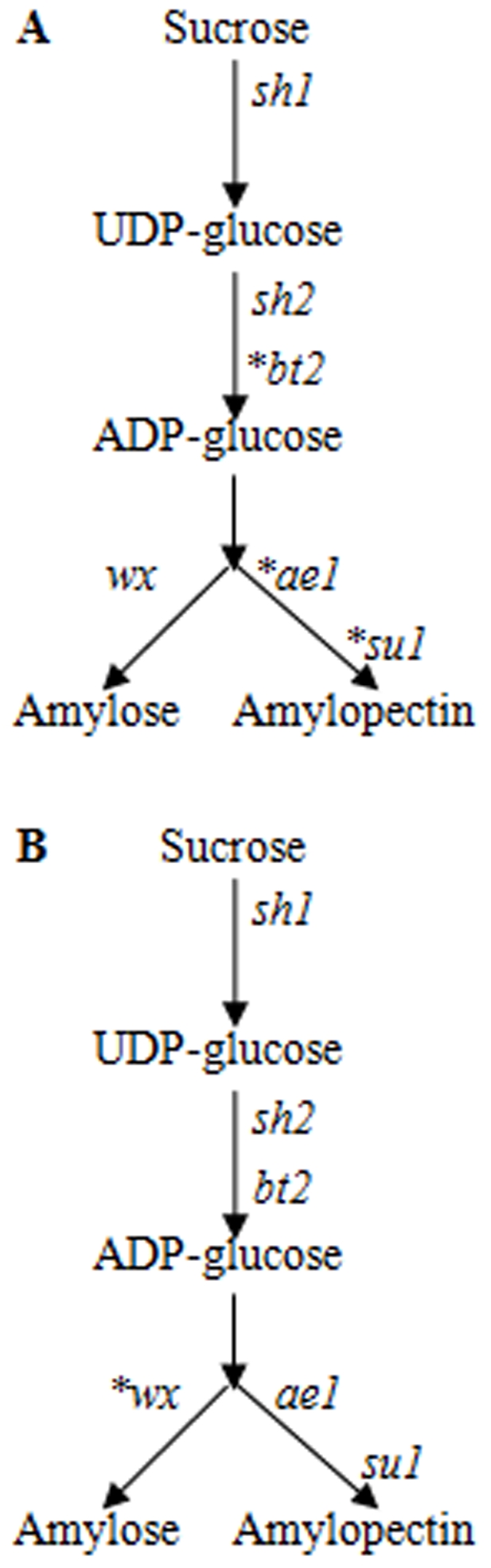
An illustration of artificial selection (domestication and/or genetic improvement) for the six sampled genes in the starch pathway. Genes with strong evidence of artificial selection are labeled with stars. A: investigation in a population of non-glutinous American maize by Whitt et al. [7]; B: investigation in a population of Chinese waxy maize in this study.

The starch pathway is critical to both the yield and quality of grains, and starches normally account for 73% of the total kernel weight [7]. Maize (*Zea mays* ssp. *mays*) has two different pathways for starch formation: one that generates branched-chain polysaccharides (amylopectin) and another that gives straight-chain polysaccharides (amylose) from a common substrate (Figure 1). The starch of normal or non-glutinous maize contains about 25% amylose, and the remainder is amylopectin. However, glutinous maize, which was first found in China in 1909 [8], produces only a small amount of amylose. Chinese waxy maize is believed to have been improved from domesticated non-glutinous maize, which was introduced into China from the New World about 500 years ago [9], [10] and is still popular as a foodstuff in China and other East Asian countries. The pathway of starch synthesis in the cereal endosperm requires many unique enzymes, and dozens of genes encoding these enzyme isoforms have been identified so far [11]. Due to its agronomic importance, great effort must have been made in artificial selection in the starch pathway in maize and other cereals during domestication and subsequent genetic improvement. Whitt et al. [7] first investigated genetic diversity and selection in the maize starch pathway by comparing six key genes that are known to play major roles in this pathway between populations of non-glutinous maize inbreds and their wild progenitor, *Z. may* ssp. *parviglumis*. The six genes were *amylose extender1* (*ae1*), *brittle2* (*bt2*), *shunken1* (*sh1*), *shrunken2* (*sh2*), *sugary1* (*su1*) and *waxy* (*wx*) (Figure 1). Their results suggested that at least three genes (*bt2*, *ae1* and *su1*) have experienced significant selective pressure of domestication and/or genetic improvement to influence the yield and/or grain quality in maize. In their investigation, similar to the results in other previous studies [12], [13], no positive selection was detected in the *wx* gene (Figure 1A).

However, recent studies [9], [14] have detected significant positive selection on *wx* in Chinese waxy maize, suggesting that strong improvement might have acted on the mutation genotype to maintain the glutinous phenotype. As mentioned above, Chinese waxy maize is believed to have arisen from the subsequent improvement of non-glutinous domesticated American maize [9], [10] and therefore provides a unique model for investigating genetic selection after the domestication of maize. In this study, six key genes in the starch pathway were investigated in Chinese waxy maize, and we sought to determine the changes in genetic diversity and selective signatures in the maize starch pathway during an improvement event.

## Results

### Two independent origins of Chinese waxy maize

**Figure 2:**
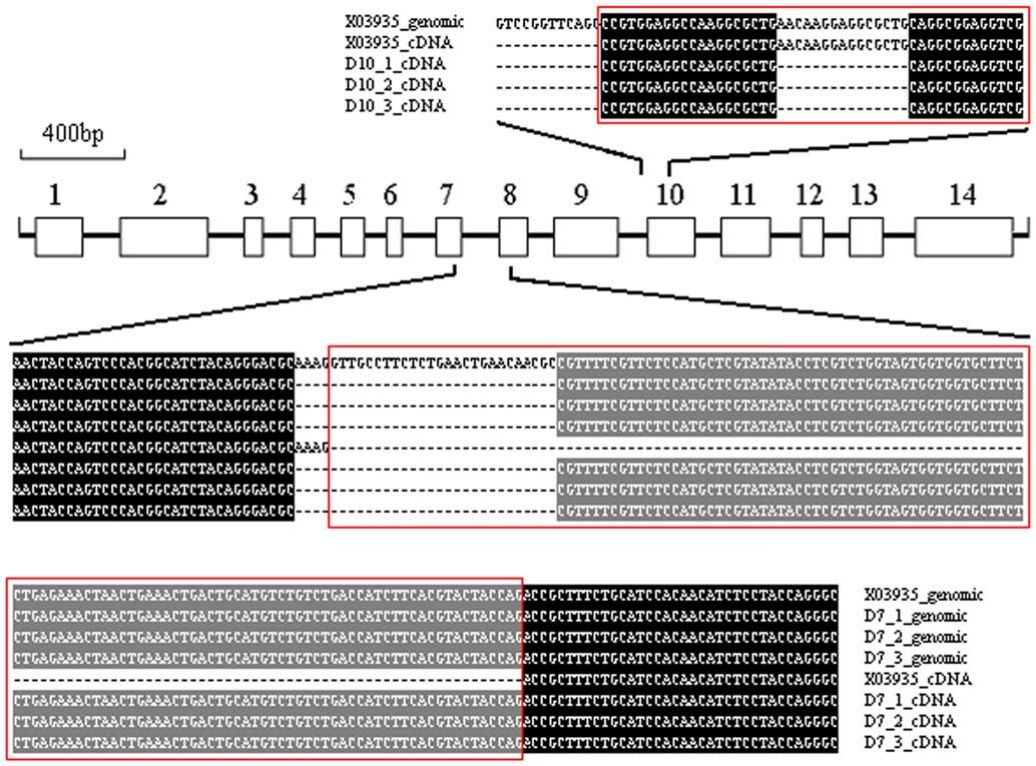
Genotyping of *wx* in Chinese waxy maize. Two deletions at the exon 7-intron 7 junction (D7) and exon 10 (D10) of *wx* are shown. Three cDNA sequences from accessions of each mutation are aligned with sequences from a reference non-glutinous maize (X03935) and intron 7 and exon 10 are boxed.

**Figure 3:**
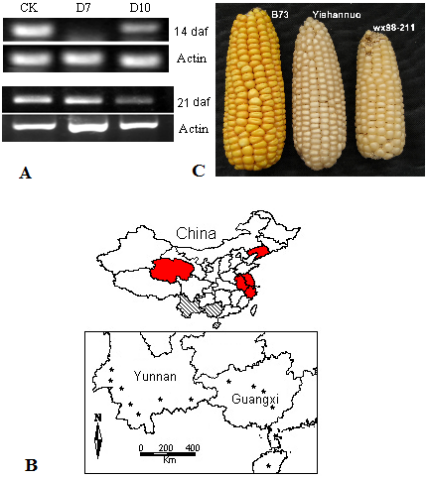
Expression and distribution of the *waxy* gene in Chinese waxy maize. A: *wx* expression in the developed seeds at different days after flowering (daf) in two *wx* mutation accessions (D7: Chiqibainuo; D10: Four-row Wax; CK: B73). B: Geographical distribution of the sampled landraces. The provinces sampled are labeled and two independent *wx* mutations were found in the Yunnan-Guangxi region (D10) and the Yangtze River region (D7) of China, respectively. The sampling sites in the Yunnan-Guangxi region are shown in the box; C: Cobs of two types of *wx* mutations, D7 (wx98–211) and D10 (Yishannuo), in Chinese waxy maize.

In our previous study, partial sequences of the *wx* gene in Chinese waxy maize were determined and several deletions at the *wx* genes were identified [9]. To determine potential causative mutations of the *wx* gene in Chinese waxy maize, full-length genomic sequences of eight accessions that were randomly selected from a glutinous population that included 55 Chinese accessions (see next section) were sequenced (GenBank accession no. GQ354129–32 and EU041689–92). Sequence analysis indicated that two mutations, a 30-bp deletion at the conjunct region of exon 7 and intron 7 (termed D7) and a 15-bp deletion at exon 10 (D10) in *wx*, were observed (Figure 2). Transcriptional sequences (cDNA sequences, GQ354123–8) of three accessions with the D7 and D10 mutations were further determined, respectively. Sequence alignment indicated that the same 15-bp deletion was also observed in the mRNA sequences of D10 accessions, while a large change (whole intron 7 was not cleaved from its mRNA sequence) was observed in D7 accessions (Figure 2). These two types of mutations showed lower expression levels of the *wx* gene compared to non-glutinous maize (B73) (Figure 3A). Besides the D7 and D10 mutations, no other glutinous maize-specific mutations were found in coding regions, splice site or promoter sequences in the full-length sequences of *wx* from eight accessions (four accessions for each mutation type), which suggests that the two mutations may cause functional failure of the *wx* gene.

We further scanned the two *wx* mutations in 28 landraces of Chinese waxy maize that were collected from eight different Chinese areas (provinces). One of the two mutations was detected in each accession of the 28 landraces. Interestingly, the samples from the Yunnan-Guangxi region all possessed the D10 mutation while others from the Yangtze River region harbored D7 (Figure 3B; Table 1). The geographical distribution suggests that the D10 genotype originated from the Yunnan-Guangxi region while D7 is from the Yangtze River region. No accession with both the D7 and D10 mutations was observed in the landraces or the inbred lines (see next section), suggesting that there may have been two independent origins or genetic improvement events for Chinese waxy maize. In brief, the glutinous phenotype which is possibly due to two *wx* mutation events (D7 and D10) was selected and fixed in the corresponding local populations of Chinese waxy maize since maize was introduced into China (two example cobs with the D7 and D10 genotypes are shown in Figure 3C).

**Table 1:** Summary of sampled accessions of Chinese waxy maize.

Accession name	Type	AAC (%)#	Mutation¶	Origin*
Tengchonghuangnuo	Landrace	0.94(0.09)	D10	1
Menghainuobaogu	Landrace	1.37(0.35)	D10	1
Menghaizinuo	Landrace	8.80(0.26)	D10	1
Luximangshiheinuo	Landrace	3.58(0.52)	D10	1
Danuobaogu	Landrace	1.87(0.87)	D10	1
Nuobaogu	Landrace	3.52(0.26)	D10	1
Nuobaogu	Landrace	0.70(0.26)	D10	1
Zaonuobaogu	Landrace	1.25(0.87)	D10	1
Qiaojiabainuo	Landrace	6.47(0.09)	D10	1
Yishannuo	Landrace	4.61(0.07)	D10	1
Luochengnuo	Landrace	1.68(0.09)	D10	1
Fengshannuo	Landrace	1.74(0.17)	D10	1
Lancangnuobaogu	Landrace	2.73(0.69)	D10	1
Yongdebainuoyumi	Landrace	4.20(0.52)	D10	1
Lvchunnuobaogu	Landrace	1.62(0.17)	D10	1
Jinghongxiaozhenuo	Landrace	3.46(0.17)	D10	1
Maguanbaogu	Landrace	0.82(0.26)	D10	1
Gongshannuobaogu	Landrace	4.38(0.43)	D10	1
Four-row Wax	Landrace	3.40(0.26)	D10	2
Chiqibainuo	Landrace	4.51(0.26)	D7	3
Hangshuangxinuo	Landrace	1.50(0.17)	D7	4
Jiangsunongpin J-4	Landrace	1.44(1.13)	D7	4
Jiangsunongpin J-1	Landrace	2.54(0.43)	D7	4
Huaizinuo	Landrace	3.46(0.00)	D7	4
Heibangzhi	Landrace	2.73(0.17)	D7	4
Shenggongbainuo	Landrace	2.91(0.09)	D7	4
Sichuanchangnuo	Landrace	3.15(0.43)	D7	4
Qiong MHS	Landrace	2.42(1.30)	D10	4
N23-16-2-2-1	Inbred	4.87(0.09)	D7	3
N11-16-1-1-1-1	Inbred	4.69(0.17)	D7	3
N26-1-1-2-2	Inbred	0.76(0.17)	D7	3
N22-4-3-2-1	Inbred	1.62(0.52)	D7	3
CN9-5-1	Inbred	0.70(0.26)	D7	3
N32-6-3-3	Inbred	4.14(0.09)	D7	3
622078-CN-78	Inbred	0.70(0.43)	D10	4
N06-24	Inbred	1.13(0.00)	D10	4
DQ65	Inbred	1.80(0.09)	D10	4
DQ55	Inbred	1.99(0.01)	D10	4
BAI-SN	Inbred	1.68(0.96)	D10	4
BTN-WX	Inbred	3.34(0.87)	D10	4
SP1	Inbred	1.56(0.09)	D10	4
622016-ZCN-2	Inbred	1.50(0.17)	D7	4
622105-CN-106	Inbred	3.46(0.17)	D7	4
622141-CN-142	Inbred	1.50(0.17)	D7	4
622147-CN-148	Inbred	0.45(0.09)	D7	4
622201-CN-203	Inbred	0.94(0.09)	D7	4
622244-CN-46	Inbred	1.74(0.00)	D7	4
622219-CN-21	Inbred	1.99(0.17)	D7	4
622023-WX98-211	Inbred	5.30(0.35)	D7	4
622033-CN-33	Inbred	1.56(0.26)	D7	4
WX-MEINUO8	Inbred	1.99(0.87)	D7	4
613177-CN-9656	Inbred	4.32(0.52)	D7	4
613159-CN-H16	Inbred	2.79(0.26)	D7	4
613109-CN-T7-1	Inbred	2.85(0.69)	D7	4
622031-CN-36	Inbred	2.30(0.09)	D7	4

#Apparent amylose content (standard error).

¶A 30-bp deletion at the junction of exon 7-intron 7 (D7) and a 15-bp deletion at exon 10 (D10) in the *wx* gene. Also see Figure 2.

* 1. Institute of Crop Science, Chinese Academy of Agricultural Sciences; 2. Institute of Genetics and Developmental Biology, Chinese Academy of Sciences; 3. Institute of Crop Science, Zhejiang University; 4. Institute of Crop Science, Zhejiang Academy of Agricultural Sciences.

### Nucleotide variation at the starch loci

To compare the changes in genetic variation at the six key starch loci [7] of Chinese waxy maize to those in non-glutinous domesticated American maize, 55 accessions, including 28 landraces and 27 inbred lines which represent a broad range of the genetic diversity of Chinese waxy maize, were selected (Table 1). Low apparent amylose contents (<7%) have been seen in the waxy accessions. On average, genomic segments of over 1000 bp for the six starch genes and sequences of about 600 bp for six neutral genes were determined from each Chinese waxy accession. Overall, more than 1 Mb of sequences (including full-length genomic and cDNA sequences) from Chinese accessions were determined in this study and deposited in GenBank. All of the sequences of non-glutinous maize came from Whitt et al. [7] and Tenaillon et al. [15], who sampled their accessions from America.

**Figure 4:**
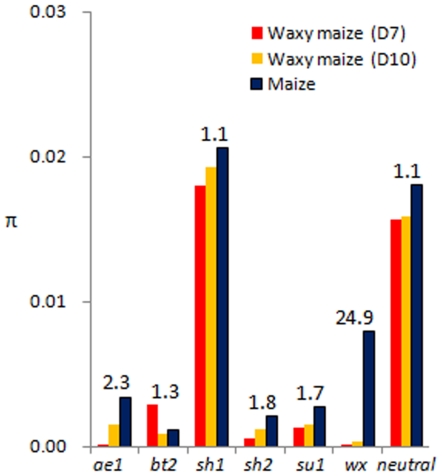
Comparison of silent diversity of the six starch genes in Chinese waxy maize and non-glutinous American maize. The numbers above the bars indicate the fold-reduction in diversity between non-glutinous maize [7] and one (D10) of the two independently domesticated subpopulations (D7 and D10) of Chinese waxy maize. Neutral refers to the average of six nonselected genes (*adh1*, *an1*, *bz2*, *csu1138*, *csu1171*, *glb1*) as reported by Tenaillon et al. [15].

A reduction in diversity of over 15% (average 16.6% and 19.1% for D10 and D7 subgroups in total sites of *π*) was found between Chinese waxy maize and non-glutinous maize based on the six neutral genes (Table 2), which suggested a selective bottleneck in improved Chinese waxy maize. A greater loss of genetic diversity (29.6% and 37.8%) was seen in the six starch genes in Chinese waxy maize compared to non-glutinous maize, and improvement selection combined with a demographic effect should have contributed to this loss (see next section for details). The extent of the reduction in diversity between Chinese waxy maize and non-glutinous maize varied among the six starch genes (Figure 4; Table 2). The fold-reduction in diversity between one subpopulation (D10) of Chinese waxy maize and non-glutinous maize is shown in Figure 4, which indicates that a more significant reduction in diversity was generally seen in the six starch genes in the other subpopulation (D7). Apparently, *wx* experienced the greatest reduction (24.9- and 53.2-fold in the D10 and D7 subgroups, respectively) in Chinese waxy maize relative to non-glutinous maize while *sh1* apparently retained its diversity (1.1-fold) in Chinese waxy maize. Four other genes showed a 1.3- to 2.3-fold reduction in diversity in Chinese waxy maize compared to non-glutinous maize. The results indicate that Chinese waxy maize experienced a genetic bottleneck during its improvement comparable to that in non-glutinous maize, which had a 1.2- to 6.2-fold reduction in diversity compared to its wild progenitor *Z. may* ssp. *parviglumis* [7], and to rice (*O. sativa*), which retained only 15–40% of the diversity of its progenitor *O. rufipogon* [16].

**Table 2:** Summary of nucleotide diversity.

Phenotype	Locus	Population*	*n*	Sites	Diversity (π×10^−3^)	
					Total	Silent
Glutinous maize						
Starch pathway	*ae1*	Glutinous-D7	23	831	0	0
		Glutinous-D10	22	830	2.04	1.47
	*bt2*	Glutinous-D7	26	1,476	2.86	2.94
		Glutinous-D10	25	1,481	1.33	0.89
	*sh1*	Glutinous-D7	25	1,603	9.89	17.98
		Glutinous-D10	26	1,653	10.05	19.32
	*sh2*	Glutinous-D7	25	1,010	1.03	0.60
		Glutinous-D10	22	959	1.60	1.15
	*su1*	Glutinous-D7	28	840	1.02	1.26
		Glutinous-D10	27	891	1.29	1.55
	*wx*	Glutinous-D7	28	733	0.38	0.15
		Glutinous-D10	25	761	0.72	0.32
	Average	Glutinous-D7	26	1,082	2.50	3.77
		Glutinous-D10	25	1,096	2.83	4.10
Reference loci						
	Average	Glutinous-D7	27	599	10.22	15.70
		Glutinous-D10	24	615	10.53	15.84
Non-glutinous maize†						
Starch pathway	*ae1*	Non-glutinous	32	697	2.66	3.41
	*bt2*	Non-glutinous	32	1,409	1.16	1.16
	*sh1*	Non-glutinous	32	1,695	10.72	20.64
	*sh2*	Non-glutinous	32	1,036	2.05	2.06
	*su1*	Non-glutinous	32	995	2.34	2.69
	*wx*	Non-glutinous	32	677	5.21	7.98
	Average	Non-glutinous	32	1,085	4.02	6.32
Reference loci						
	Average	Non-glutinous	25	589	12.63	18.04

* Populations include two subpopulations from two independent origins (D7 and D10) of Chinese waxy maize and a non-glutinous American population [7].

† Summary of six starch loci by Whitt et al. [7] and the reference loci (*adh1*, *an1*, *bz2*, *csu1138*, *csu1171* and *glb1*) reported by Tenaillon et al. [15].

### Positive selection on the starch loci

Three genes have been identified as candidate genes of domestication and/or improvement by Whitt et al. [7] based on the HKA (*bt2* and *su1*) and Tajima's *D* (*ae1*) tests in a non-glutinous maize population (Table 3). An additional test used in this study, coalescent simulation (CS) analysis, supports their conclusion (Table 3). The CS approach incorporates summary statistics to estimate the duration and severity of the bottleneck based on data from reference genes and then tests whether the loss of diversity at a candidate locus is too great to be explained by demographic effects alone [17], [18]. Therefore, this analysis does not rely on the standard neutral model and is different from the Tajima's *D* and HKA tests, the results of which can be influenced by the demographic history [6]. Signatures of positive selection for *ae1* and *su1*, but not *bt2*, as revealed by the CS test, have also been detected in two subpopulations of Chinese waxy maize. All three tests identified positive selection on the *wx* gene in both glutinous subpopulations, but not in a non-glutinous subpopulation, which suggests that strong post-domestication improvement has acted on the locus in the Chinese waxy population. The sharp reduction in polymorphism at this locus in the glutinous population compared to the non-glutinous population is consistent with the neutral test results. No significant change in selective force was observed between glutinous and non-glutinous populations for the two other genes (*sh1* and *sh2*). A similar result in a neutrality test was obtained in Chinese waxy maize for the six starch genes based on their silent sites.

**Table 3:** Results of the tests for selection.

Locus	Population	*n*	*S*	Tajima's *D*	*P* value in HKA	*P* value in CS
*ae1*	Glutinous-D7	23	0	/	/	<0.001
	Glutinous-D10	22	9	−1.05	0.100	0.002
	Non-glutinous	32	17	−1.89*	0.043	0.002
*bt2*	Glutinous-D7	26	16	0.02	0.021	0.640
	Glutinous-D10	25	10	−1.03	0.001	0.245
	Non-glutinous	32	5	0.86	<0.001	0.046
*sh1*	Glutinous-D7	25	59	0.06	0.127	0.907
	Glutinous-D10	26	57	0.43	0.785	0.843
	Non-glutinous	32	52	1.51	0.942	0.644
*sh2*	Glutinous-D7	25	9	−1.82*	0.021	0.951
	Glutinous-D10	22	12	−1.87*	0.079	0.991
	Non-glutinous	32	14	−1.28	0.115	0.993
*su1*	Glutinous-D7	28	5	−0.92	0.004	0.001
	Glutinous-D10	27	8	−1.38	0.009	0.002
	Non-glutinous	32	8	0.52	0.014	0.002
*wx*	Glutinous-D7	28	4	−1.89*	<0.001	0.012
	Glutinous-D10	25	6	−1.95*	<0.001	0.022
	Non-glutinous	32	20	−1.00	0.272	0.168

Population, two subpopulations of Chinese waxy maize from independent origins (D7 and D10) and a non-glutinous American population [7]; *n*, number of sampled sequences; *S*, number of segregating sites; HKA test, *P* value of candidate gene by a multiple-locus HKA test against four neutral genes by the maximum cell value test; CS, coalescent simulation of domestication test [17]. *, *P*<0.05.

Due to the apparent geographic structure of our samples (i.e. two subgroups of D7 and D10), neutrality tests using a pooled set of our samples failed to find strong evidence of positive selection on *wx* (data not shown). For example, Tajima's *D* values were elevated towards positive values when a pooled sample was used. Genetic variants tend to be over-estimated for a pooled sample that came from distinct subgroups (such as D7 and D10 in this study), resulting in an excess of intermediate frequency variants, and Tajima's *D* statistic can be elevated toward a positive value [6].

**Figure 5:**
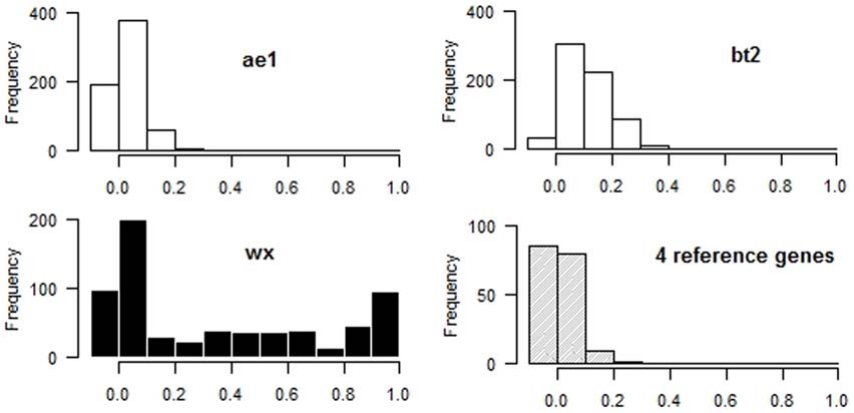
Distribution of the *Fst* values between Chinese waxy maize and non-glutinous American maize in three starch-pathway genes (*ae1*, *bt2* and *wx*) and a reference set of genes. The non-glutinous maize is based on Whitt et al. [7] and Tenaillon et al. [15].

As shown above, positive selection has been detected in Chinese waxy maize for several genes. We sought to determine whether the signatures are footprints of the improvement of Chinese waxy maize. Genes with unusually strong differentiation between populations, for example, *Phr1* for grain discoloration in rice [2], are often suggested to be under population-specific selection [18]. We calculated the extent of population differentiation as estimated by the *Fst* statistic [19] between Chinese waxy maize and non-glutinous maize for the six starch genes. For comparison, a data set containing the four neutral reference genes was used as a control. The distributions of the *Fst* statistic between the glutinous (D7 subgroup) and non-glutinous populations are shown in Figure 5. High *Fst* values close to 1 indicate strong genetic differentiation between populations, and a low *Fst* near 0 suggests homogeneity [2]. *wx* was shown to have a high peak near *Fst* = 1 (Figure 5), while the other genes (e.g. *ae1* and *bt2* in Figure 5) and the reference data set did not have a similar peak when we compared the glutinous and non-glutinous populations. On the other hand, the *Fst* profile of *wx* was different from those of other genes (all with *P*<2.2×10^−16^ by the one-sided Kolmogorov-Smirnov test). These results suggest that, among the six starch genes, *wx* is the only unusually differentiated gene between the glutinous and non-glutinous populations.

## Discussion

### Shift in the selection target between *wx* and *ae1* during improvement

Two key genes (*wx* and *ae1*) decode granule-bound starch synthase and starch branching enzyme, which catalyze ADP-glucose into amylose and amylopectin, respectively. The genes that encode the branching enzyme (such as *ae1* and *su1*) have experienced strong positive selection during the domestication and/or improvement of non-glutinous maize (Figure 1A) [7]. Due to their roles in such an important agronomic trait, it is easy to understand why these genes became targets of domestication and/or improvement during the rise of maize. However, for subsequent directional improvement for an agronomic trait (e.g. glutinous or an almost 100% amylopectin content among starch compounds), the selection targets seem to have shifted to the *wx* gene, for which loss-of-function results in a dramatic reduction in amylose production and thus an increase in amylopectin. There is clear evidence for a shift in the selection target. First, strong directional selection led to a sharp reduction in the diversity of *wx* during the improvement of Chinese waxy maize. Second, Whitt et al. obtained evidence for positive selection in *ae1* but not *wx* during the domestication and improvement of a non-glutinous maize population using Tajima's *D* test [7]. A lack of positive selection on *wx* has also been reported by other studies; e.g. [12], [13]. In contrast, significant negative values for Tajima's *D* were found in this study for *wx* but not *ae1* in the glutinous population. Our results for *wx* are consistent with the results of Tajima's *D* reported for other glutinous maize populations [9], [14] and are also consistent with another independent investigation of linkage disequilibrium (LD) in glutinous maize by a Korean group [20], who compared single-nucleotide amplified polymorphisms (SNAPs) of the six key kernel starch synthesis genes between glutinous and non-glutinous maize. They detected significant LD with *wx* but not *ae1* in the glutinous group and significant LD with *ae1* but not *wx* in the non-glutinous group. Directional or positive selection tends to increase LD in target genes [6]. Third, *wx* and *ae1* presented two different distribution profiles of the *Fst* values between glutinous and non-glutinous maize (Figure 5), which suggests that *wx* but not *ae1* shows unusual differentiation between the two populations.

In previous studies, significant neutral test results were found in *sh2* in both maize and *Z. may* ssp. *parviglumis*, indicating that selection might have occurred before the divergence of maize from its wild progenitor, i.e., it has been constrained by natural selection but has escaped domestication [7], [21]. Although significant departures from neutrality according to Tajima's *D* and HKA tests were observed for *sh2* and *bt2* in Chinese waxy maize (Table 3), it is reasonable to believe that no additive positive selection in *sh2* and *bt2* occurred during the improvement of Chinese waxy maize, since Tajima's *D* is negatively skewed in maize [22], and the reductions in diversity and *Fst* profiles are not distinct from those in the neutral genes.

Taken together, our results indicate that *wx* is the only post-domestication-selected gene among the six starch genes in Chinese waxy maize (Figure 1B), and suggest that an agronomic trait can be quickly improved into a target with changes in the selection target among genes in a pathway. Meanwhile, improvement seems to target only one key gene (e.g. *wx*), which basically can change maize into the target phenotype. In many cases where causal mutations have been identified, such as non-shattering in rice cultivars, free threshing or naked seeds in barley, and naked grains of maize, a single mutation primarily controls a domestication transition, i.e., one gene for one domestication trait [23]. Thus, it is reasonable to speculate that in most cases a single gene can play a critical role in a key improvement transition.

### Mutation and evolution of waxy maize

Since their recessive mutations are expressed in an easily identifiable nonlethal phenotype, both glutinous and sweet maize have been easy targets of artificial selection. At least five independent mutations of s*u1* have been involved in the origin of sweet maize and the role of recurrent mutation in crop evolution has been highlighted [24], [25]. The present study showed that two origins (D7 and D10) of Chinese waxy maize were improved independently in the Yunnan-Guangxi region and the Yangtze River region of China. Moreover, some Chinese waxy accessions without the two *wx* deletions were also identified (data not shown), which suggests that there may be additional origins of Chinese waxy maize. Multiple independent mutations have also been involved in the domestication and improvement of other crops; for example, *Vrs1* for the six-rowed phenotype in barley [26] and *Phr1* for grain discoloration in rice [2]. Taken together, our results in waxy maize seem to confirm that recurrent mutation plays an important role in crop domestication and genetic improvement.

A previous investigation with glutinous rice also showed a significant negative Tajima's *D* value for the *wx* gene where a mutation in an intron 1 splice donor site is responsible for the glutinous phenotype [27]. This result is similar to ours in maize, which suggests that glutinous crops may show the same pattern of artificial selection on *wx*.

Okagaki et al. reported a common deletion in two independently derived waxy mutations of maize through ethyl methanesulfonate (EMS) mutagenesis [28]. The deletion is the same 30-bp deletion at the conjunct region of exon 7 and intron 7 (i.e. D7) as in Chinese waxy maize. These results suggest that the D7 location seems to be a mutation hotspot which was chosen as a selection target during the improvement of Chinese waxy maize.

## Materials and Methods

### Sampling

Fifty-five accessions were selected from a wide range of geographical locations in China to represent a broad range of genetic diversity within landraces and inbreds of Chinese waxy maize (*Zea mays* ssp. *mays*) (Table 1). Their apparent amylose contents were estimated following Fan et al. [9]. *Tripsacum dactyloides* seeds were kindly provided by GRIN Plant/Database Records, USDA-ARS.

### DNA isolation and sequencing

Genomic DNA of Chinese waxy maize was isolated from 14 d leaves using the CTAB protocol with minor modification. PCR conditions were optimized for each primer pair. PCR products were purified and sequenced directly with each oligo nucleotide primer (Invitrogen). For the 5′ end regions with high GC contents at the *wx* gene, LA Taq™ with a GC Buffer kit (Takara) was used. 750–1700-bp segments of six key genes (*bt2, sh1, sh2, ae1, su1, wx*) in the starch pathway [7] and six neutral loci (*adh1, an1, bz2, csu1171, csu1138, glb1*) [15] were determined for the 55 Chinese accessions (Table 1). Of the 55 accessions, the full-length genomic sequences of *wx* were further sequenced for eight (Yishannuo, Qiaojiabainuo, Chiqibainuo, Four-row Wax, Lancangnuobaogu, Qiong MHS, Jiangsunongpin J-4, 622078-CN-78). Primers (Table S1) were designed based on genomic sequences (X03935) of non-glutinous maize using Primer3 [29]. Each accession was PCR-amplified from both directions and their sequences were assembled using Bioedit [30]. Unique single-base-pair variants (singletons) were manually checked by examining their raw chromatogram peaks using Chromas software (http://www.technelysium.com.au/chromas.html). All sequence data from this article were deposited in GenBank under accession numbers GQ353472-GQ354132 and EU41689-EU41692. Sequences of non-glutinous American maize reported by Whitt et al. [7] and Tenaillon et al. [15] were downloaded from GenBank.

### Gene expression and cDNA sequencing

RNA was extracted from maize developing endosperm at 14, 21 and 28 days after *flowering* using TaKaRa RNAiso™ Reagent. The cDNA was synthesized from 3 µg of total RNA using an oligo-dT primer at 42°C with a PrimeScript™ 1st Strand cDNA Synthesis Kit (TaKaRa) in a total volume of 20 µl. A 1-µl aliquot of single-stranded cDNA was used as a template for RT-PCR amplification in a total volume of 20 µl. Primer pairs for wx2182 and actin (Table S1) were used to analyze *wx* gene expression in glutinous (Qiaojiabainuo and Chiqibainuo) and non-glutinous accessions (B73). Thirty and 35 cycles of RT-PCR were performed using taq plus DNA polymerase (Sangon) and the amplification products were detected using 1.5% agarose gel. The full-length cDNAs of *wx* in six accessions (Yishannuo, Four-row Wax, Chiqibainuo, Qiaojiabainuo, N11-16-1-1-1-1, 622023-WX98-211) were determined. PCR products were purified and sequenced directly.

### Data analysis

ClustalW [31] was used for multiple alignments with manual refinement. DnaSP software [32] was used to calculate the number of segregating sites (*S*), the number of haplotypes (*h*), and the average proportion of pairwise nucleotide differences per nucleotide site (*π*) [33], and also to provide a neutral test of Tajima's *D* [34]. HKA tests [35] were conducted using HKA software (http://lifesci.rutgers.edu/~heylab/HeylabSoftware.htm#HKA). Insertions/deletions (indels) were excluded from our analysis and total sites were used for the two neutral tests, with *T. dactyloides* as an outgroup. R scripts were kindly provided by Tian Tang [2] and used to calculate *Fst* statistics as described in the literature [19] with minor modifications. The distributions of *Fst* values for each locus in the starch pathway were obtained by 100 bootstrap resampling. Coalescent simulation analysis was performed to determine if a gene was a potential target of positive selection with regard to demographic factors. We used the bottleneck model (model 1) described by Eyre-Walker et al. [36] and the parameters were calculated as described by Tenaillon *et al*. [17]. *4Nc_hud87_* was estimated for each gene as a recombination parameter. The severity of the bottleneck (*k*), defined as the ratio of the population size during the bottleneck (*N*_b_) to the duration of the bottleneck (*d*), was set at 1.2, since in maize it has been estimated by several groups to range from 2.0 [22] to 4.5 [17]. A total of 10,000 simulations were carried out for each gene based on the coalescent model described above. A gene was considered to be a potential target of selection during domestication if the segregating sites of the tested gene comprised <97.5% of the segregating sites of simulations [22].

## Supporting Information

Table S1 Primer pairs used in this study
(0.05 MB DOC)
