# Supplementary material for: Post-Domestication Selection in the Maize Starch Pathway
Source: PLoS One. 2009 Oct 27;4(10):e7612. doi: 10.1371/journal.pone.0007612 (PMC2762603; doi:10.1371/journal.pone.0007612)
Supplement: Table S1 — Primer pairs used in this study (0.05 MB DOC) [file pone.0007612.s001.doc]

**Table S1 Primer pairs used in this study**

| primer code | 5'- Forward primer -3' | 5'- Reverse primer -3' | product size (bp) |
| --- | --- | --- | --- |
| Wx2182 | CAGGGGATGCGATACGGA | CATTACACTAGCACAAGCAAGCAG | 483 |
| Actin | CACTACGACTGCTGAGCGAGAA | CCAATGAAGGATGGCTGGAATA | 204 |
| Ae1 | CGCTCGTCTCCGTGCTAT | ATTCTCCCTCACTCCCCAAC | 1015 |
| Bt2 | TCTCCAATCCCCTCTACGTG | TCCGCACCCATTAGTAAACTG | 1850 |
| sh1-3 | CAAGGCCTGGTGTCTGGGATTAC | AGGTCGAGAAGCAAGTGGAGTGTG | 830 |
| Sh1-4 | CATCCCTGAGAAAGGCAGAG | GCGAATGATGTCTGTGTGCT | 886 |
| Sh1-5 | GTTGCCTGATGCTGCTGGGACTAC | GTGAACGCGATGTGGGACTC | 743 |
| Sh2 | AACCAAAGGGTGCTGATTTG | TGCCTGGTGTTGAAATGGTA | 1247 |
| Su1 | GATGGATTTGCTGGTGCTTT | GGATGCAGGGAAAGACTGAA | 1279 |
| adh1 | GGCTCCCCTTGATAAAGTTTG | TGTGCGTGATGAACTTCTCC | 1325 |
| An1 | ATGGCAGGAAACCACAAGAC | GCCGAAGGAGAGAACAAGAA | 664 |
| bz2 | ACTACCGGCTCCCGTTTA | CCTTCTCACCACCGCATT | 1092 |
| Csu1132 | TGTGCTTGGGGTCGAGGTGTTTAG | TTTTTCCCCAAGCTTATTCATCTG | 598 |
| Csu1138 | AGCCAAAGCCAAAGCCACAACCTA | AACAACGCACACTCAAATCATACA | 499 |
| Csu1171 | GCACCGAACAGTCAATCAGA | AAAGCCATACGACGCACTTT | 724 |
| glb1 | CGCCGGGGCCGAAGAAGAAC | CGGATAAGCACGGTAAGGAGAGTA | 1108 |
| WxCDS1-743 | CGTCACATCCATCCATCGAC | TTGTCCCTGTAGTCCGTTCC | 743 |
| WxCDS16-792 | ACGTCGCAGCTCGTCGCA | GCCCTGGTAGGAGATGTTGTGG | 777 |
| WxCDS620-1673 | GCCTCAACAACAACCCAT | TTCCTCACCATCTCCTCGT | 1054 |
| WxCDS1255-2188 | GACAAGTACATCGCCGTGAA | AGCACAAGCAAGCAGCTACA | 934 |
| Wx209-1880 | AGTACCAGCACAGCACGTTG | GTACCCGTCTCCCATCTTGA | 1672 |
| Wxe3-7 | GTCTTCTTCGTGCTCTTGCC | GATGCCGTGGGACTGGTAG | 907 |
| Wxe4-9 | GTTGACCACCCACTGTTCCT | ATGAGCTCCTCGGCGTAGTA | 880 |
| Wxe7-10 | AACTACCAGTCCCACGGCATCT | CACGTCCTCCACCATCTCCAT | 902 |
| Wxe9-13 | TGCGAGCTCGACAACATCATGCG | AGGGCGCGGCCACGTTCTCC | 1344 |
| Wxe11-14 | GAGAAGTTCCCAGGCAAGGT | AGCACAAGCAAGCAGCTACA | 931 |
